# When TNF‐α becomes a matter of the heart

**DOI:** 10.1111/jdv.20866

**Published:** 2025-09-26

**Authors:** Wolf‐Henning Boehncke

**Affiliations:** ^1^ Division of Dermatology and Venereology Geneva University Hospitals Geneva Switzerland

Physicians across several specialties are aware of guidelines recommending avoidance of TNF‐α inhibitors (TNF‐i) when treating patients for immune‐mediated inflammatory diseases (IMIDs) who also suffer from advanced heart failure (HF). This is primarily based on an FDA report on early post‐marketing surveillance data^1^ and clinical trials failing to show a clinical benefit in HF.^2^ Remarkably, the meta‐analysis by Galajda et al. published in this issue of the JEADV indicates that TNF‐i‐treated IMID patients neither have an increased risk for developing de novo HF, nor a statistically significant risk enhancement for worsening HF.^3^

The evidence generated by Galajda et al. is strong: Methodologically solid, they distinguish between worsening and de novo HF, considering randomized trials as well as non‐randomized observational studies. Of note, another meta‐analysis showed even a (statistically non‐significant) risk‐reducing effect of TNF‐i therapy.^4^ It is confusing that companies invested heavily to study TNF‐i for the treatment of HF only to learn from the FDA that this may be harmful, which in the end may not be the case.

A closer look at what is generally accepted regarding the role of TNF‐α in HF helps to better understand the discussion^5^: HF is a condition in which the heart either cannot pump enough blood or can do so only at the cost of increased filling pressures. It is triggered by a wide range of pathophysiologic changes, including infarction, pressure or volume overload, or metabolic dysregulation. Regardless of the underlying aetiology, inflammatory signalling cascades are activated which promote dysfunction and progressive remodelling of the myocardium. The best‐studied cytokine in this context is TNF‐α. Increased myocardial expression of TNF‐α has been consistently documented in experimental models and in patients, with several lines of evidence pointing towards a causative role. TNF‐α‐mediated adverse remodelling and HF progression may involve effects on cardiomyocytes, macrophages, the extracellular matrix and endothelial cells; but it may also exert protective actions on injured or stressed cardiomyocytes (Table 1). It has been suggested that deleterious and beneficial effects may be mediated through distinct receptors (TNFR1 and TNFR2, respectively).

**TABLE 1:** Synopsis of negative and protective effects of TNF‐i in HF.

Cell type	Negative effect	Protective effect
Cardiomyocytes	Reduced contractility through perturbed calcium homeostasis Pro‐apoptotic	Formation of an alternative cytoskeletal network Improved haemodynamics through reduced intracellular calcium overload Protected mitochondrial function through modulation of reactive oxygen species and sphingolipids
Macrophages	Stimulating synthesis of additional pro‐inflammatory cytokines Reduced contractility Pro‐apoptotic Matrix‐degrading	
Fibroblasts	Matrix degradation through dysregulated matrix‐metalloproteinases	
Microvasculature	Increased permeability through modulation of endothelial cyclooxygenase‐2 Leukocyte extravasation through upregulated adhesion molecules	

There are several potential explanations for the discrepancy between the rather promising results of TNF‐i therapy for HF in animal models (and even some early clinical studies) on one hand and the disappointing large clinical studies on the other hand:

- In human patients, the well‐described protective effects of TNF‐α may be more prominent compared to the deleterious effects.
- HF is heterogeneous, and only (yet to be defined) subpopulations may benefit.
- Dose‐dependent effects may contribute to the complexity of the scenario, as failing hearts may require an optimal TNF‐α level to preserve function.

Taken together, TNF‐α has both negative and protective effects in HF. Besides, the trials studying etanercept for HF were stopped prematurely due to lack of clinical benefit, which is not the same as adverse events (although an infliximab trial showed increased HF hospitalizations). Finally, the above‐mentioned FDA report is based on relatively weak evidence. It is therefore not too surprising that two meta‐analyses do not support the guideline recommendations. The latter, however, refer to patients with ‘advanced’ HF only, in whom TNF‐i may well be problematic (see above).

In daily practice of dermatologists, this discussion becomes relatively academic, as patients with advanced HF are usually followed by a cardiologist, with whom an individualized treatment strategy can be discussed. Besides, TNF‐i can often be avoided, as there are alternative (often more effective and safer) targeted therapies available to treat IMIDS in dermatology, putting the need to revise the respective guidelines into perspective.

## CONFLICT OF INTEREST STATEMENT

W‐HB has received honoraria as a speaker and/or advisor from Abbvie, Almirall, Eli Lilly, Johnson & Johnson, Leo, Novartis, and UCB.

## Data Availability

Data sharing is not applicable to this article as no new data were created or analysed in this study.
